# Antimicrobial activity of nature-inspired molecules against multidrug-resistant bacteria

**DOI:** 10.3389/fmicb.2023.1336856

**Published:** 2024-01-22

**Authors:** Mohamad Hamad, Farah Al-Marzooq, Vunnam Srinivasulu, Ashna Sulaiman, Varsha Menon, Wafaa S. Ramadan, Raafat El-Awady, Taleb H. Al-Tel

**Affiliations:** ^1^Sharjah Institute for Medical Research, University of Sharjah, Sharjah, United Arab Emirates; ^2^College of Health Sciences, University of Sharjah, Sharjah, United Arab Emirates; ^3^College of Medicine and Health Sciences, UAE University, Al Ain, United Arab Emirates; ^4^College of Pharmacy, University of Sharjah, Sharjah, United Arab Emirates

**Keywords:** MRSA – methicillin-resistant *Staphylococcus aureus*, antimicribial, AMR (antimicrobial resistance), multidrug resistance (MDR), *Acinetobacter baumannii*

## Abstract

Multidrug-resistant bacterial infections present a serious challenge to global health. In addition to the spread of antibiotic resistance, some bacteria can form persister cells which are tolerant to most antibiotics and can lead to treatment failure or relapse. In the present work, we report the discovery of a new class of small molecules with potent antimicrobial activity against Gram-positive bacteria and moderate activity against Gram-negative drug-resistant bacterial pathogens. The lead compound SIMR 2404 had a minimal inhibitory concentration (MIC) of 2 μg/mL against methicillin-resistant *Staphylococcus aureus* (MRSA) and vancomycin-intermediate *Staphylococcus aureus* (VISA). The MIC values against Gram-negative bacteria such as *Escherichia coli* and *Actinobacteria baumannii* were between 8–32 μg/mL. Time-kill experiments show that compound SIMR 2404 can rapidly kill tested bacteria. Compound SIMR 2404 was also found to rapidly kill MRSA persisters which display high levels of tolerance to conventional antibiotics. In antibiotic evolution experiments, MRSA quickly developed resistance to ciprofloxacin but failed to develop resistance to compound SIMR 2404 even after 24 serial passages. Compound SIMR 2404 was not toxic to normal human fibroblast at a concentration of 4 μg/mL which is twice the MIC concentration against MRSA. However, at a concentration of 8 μg/mL or higher, it showed cytotoxic activity indicating that it is not ideal as a candidate against Gram-negative bacteria. The acceptable toxicity profile and rapid antibacterial activity against MRSA highlight the potential of these molecules for further studies as anti-MRSA agents.

## Introduction

Antimicrobial resistance (AMR) among bacterial pathogens is a silent pandemic and a challenge to global public health (Prestinaci et al., 2015; Hamad et al., 2019). A recent study estimates that AMR was behind 4.95 million deaths in 2019 (Murray, 2022). Around three-quarters of these deaths were attributed to only six bacterial pathogens which included *Escherichia coli*, *Staphylococcus aureus*, *Klebsiella pneumoniae*, *Streptococcus pneumoniae*, *Acinetobacter baumannii*, and *Pseudomonas aeruginosa* (Murray, 2022). These multidrug-resistant (MDR) bacterial pathogens were also classified by the World Health Organization (WHO) as a priority for antimicrobial development and discovery (WHO, 2017; Tacconelli et al., 2018). Along with surveillance and antibiotic stewardship, the discovery of new antimicrobial agents is urgently needed to mitigate the global challenge of AMR (Schrader et al., 2020).

Methicillin-resistant *Staphylococcus aureus* (MRSA) is a Gram-positive bacterial pathogen that causes a wide array of infections that are often difficult to treat (Gardete and Tomasz, 2014; Turner et al., 2019). Antibiotics used for MRSA treatment include vancomycin, daptomycin, and linezolid; however, several MRSA strains resistant to these antibiotics have emerged (Gu et al., 2013; Gardete and Tomasz, 2014; Roch et al., 2017). MRSA can also form persister cells and biofilms which are tolerant to most clinically available antibiotics (Lewis, 2005). Persister cells can arise during stress conditions such as stationary phase, anaerobic conditions, biofilm, antibiotic treatment, or infections and usually have low metabolic rates (Lewis, 2005, 2010; Fauvart et al., 2011; Hamad et al., 2011; Peyrusson et al., 2020). During such conditions, bacterial cells enter a low energy state, and as most antibiotics require active metabolism for their antibacterial activity, the low metabolic rates of persisters can cause tolerance to most clinically available antibiotics (Allison et al., 2011).

In this work, we describe the antimicrobial activity of a new series of compounds with potent antimicrobial activity against MRSA including vancomycin-intermediate *Staphylococcus aureus* (VISA). These molecules were also active against *Escherichia coli* and *Actinobacteria baumannii*, albeit having higher minimal inhibitory concentrations (MICs). A representative compound, SIMR 2404, showed rapid killing activity against tested Gram-positive and Gram-negative bacteria. Furthermore, this compound was also found to have rapid killing activity against persister MRSA. In antibiotic resistance evolution experiments, MRSA failed to develop resistance to SIMR 2404, unlike the antibiotic ciprofloxacin whereby resistance can readily develop. On the other hand, *E. coli* was able to develop resistance against SIMR 2404 after several passages. Our data suggest these compounds have the potential for further studies as possible antimicrobials against MRSA.

## Materials and methods

### Bacterial strains

All bacterial strains used in this study are listed in Supplementary Table S1. Mueller-Hinton (MH) media (Biolife, Italy) was used as a growth media and was incubated at 37°C. Antibiotic resistance profiles were performed using agar disk diffusion assay according to the Clinical and Laboratory Standards Institute guidelines (Clsi, 2018b). Bacteria resistant to at least three different classes of antibiotics were considered multidrug-resistant (Magiorakos et al., 2012).

### Synthesis of compounds

The synthesis of tested compounds is detailed in our previously reported procedures (Altel et al., 2021; Srinivasulu et al., 2021). Confirmation of structures was carried out using NMR, and purity was greater than 99% as determined by HPLC (Altel et al., 2021; Srinivasulu et al., 2021). The chemical structures of compounds used in this study are presented in Supplementary Figure S1.

### Determination of minimum inhibitory concentrations

For compounds or antibiotics, the MICs were determined using two-fold dilutions in MH broth according to the microdilution method by the Clinical and Laboratory Standards Institute guidelines (Clsi, 2018a). Fresh bacteria were harvested from MH agar plates and adjusted to 0.5 McFarland (10^8^ CFU/mL) in sterile 0.85% NaCl solution. Bacteria were then diluted at 1:100 in MH broth media containing test compounds or antibiotics, at twofold dilutions, in sterile 96 well plates. Plates were then incubated aerobically at 37°C for 20 h after which growth within the wells was visually inspected.

### Time-kill experiments

Time-kill assays were conducted using the broth microdilution method and enumeration of viable cells following treatment. Exponentially grown bacteria were adjusted to 5 × 10^5^ CFU/mL and cultures were treated with two-fold serial dilutions of test compounds. Samples were removed at different time points, serially diluted in 0.85% NaCl solution, and 5–100 μL of each dilution were plated onto MH agar. Following 24 h of incubation at 37°C, colonies were counted to enumerate viable cells. Experiments were conducted in triplicates.

### Preparation of MRSA persisters and killing assays of persisters

MRSA persister cells were prepared as previously described (Hamad et al., 2022). Persister *S. aureus* was isolated by treating stationary phase bacteria with high antibiotic concentrations for 4 h (Kim et al., 2016, 2018c). MRSA-ATCC 33591 strain was used for these experiments due to its susceptibility to the antibiotics gentamicin, ciprofloxacin, and vancomycin. Stationary phase MRSA-ATCC 33591 grown in 25 mL MH broth for 16 h with shaking were washed three times with phosphate-buffered saline (PBS). The cultures were then treated for 4 h with 100 × MIC gentamicin (250 μg/mL). Following treatment, cells were washed three times with PBS, adjusted to 10^7^ CFU/mL, and then treated with the test compound or control antibiotics at indicated concentrations. At specific time points, a 50 μL sample was removed, serially diluted, and spot-plated on MH agar plates to determine viable cell counts (Kim et al., 2015). In a control experiment, exponentially growing MRSA-ATCC 33591 was treated with gentamicin at 100 × MIC to confirm that this high antibiotic concentration eradicates all non-persisters. The detection limit was 1:100 CFU/mL. Experiments were conducted in triplicates.

### Fluorescence microscopy

MRSA-ATCC 33591 was grown in MH broth to ~2 × 10^5^ CFU/mL (early exponential phase), after which 1.5 mL cultures were treated with 2 × MIC of compound SIMR 2404 (4 μg/mL) or control antibiotic ciprofloxacin (0.6 μg/mL). Treated cultures were incubated at 37°C, and at different time points; cells were harvested by centrifugation at 3,000 × g for 5 min and washed two times with sterile water. Cells were then resuspended in 50 μL of water and stained with Live/Dead nucleic acid stains (Molecular Probes, United States): SYTO 9 (5 μM) and propidium iodide (15 μM) in the dark at room temperature for 15 min. A measurement of 5 μL of the stained bacteria were then applied onto a glass slide, covered with a glass coverslip, and images were obtained using fluorescence microscope (IX73, Olympus, United States) at an excitation/emission wavelength of 480/500 nm for SYTO 9 (green) and 490/635 nm for propidium iodide (red) (Asadishad et al., 2011; Huang and Yousef, 2014). ImageJ software was used to quantify live cells (green) and dead cells (red or yellow) (National Institutes of Health, US). Experiments were conducted in triplicates.

### Multi-step resistance evolution experiment

MRSA-ATCC 33591 or *E. coli* ATCC BAA2469 was adjusted to 5 × 10^5^ CFU/mL and then exposed to sub-MIC concentrations of SIMR 2404 or control antibiotics. Ciprofloxacin and colistin were used as control antibiotics for MRSA and *E. coli*, respectively. Experiments were carried out in a 96 well-plate at a volume of 100 μL MH broth. Following static incubation at 37°C for 24 h, wells with visible bacterial growth at the highest concentration were used to inoculate the next passage at 1:100 dilution. At each sequential passage, a 10% increment of the concentration of SIMR 2404 or antibiotic was performed (for up to an 80% increase). This procedure was carried out three times for 24 consecutive days for each experiment (Lahiri and Alm, 2016). Experiments were conducted in triplicates.

### Toxicity to normal human fibroblast

The sulforhodamine B (SRB) assay was used to evaluate the cytotoxic effect of compound SIMR 2404 on the survival of normal human fibroblast cells (F180), as previously described (Ramadan et al., 2018). The anticancer drug doxorubicin was used as a positive control, while DMSO served as a negative control. Briefly, F180 cells were cultured in Dulbecco’s Modified Eagle Medium (DMEM) at a density of 7,000 cells/well in a 96-well plate for 24 h, after which cells were treated with SIMR 2404 or controls for an additional 48 h. Cells were then fixed with 50% trichloroacetic acid for 1 h at 4°C. After washing nine times using water, 0.4% SRB was used to stain cells for 30 min at room temperature. Cells were then washed with 1% acetic acid and the dye was solubilized in 200 μL of 10 mM Tris base for 10 min. The optical density (OD) was then measured at 492 nm in a microplate reader Varioskan^™^ Flash (Thermo Fisher Scientific, Waltham, MA, United States). Experiments were conducted in triplicates.

## Results

### Identification of small molecules with antimicrobial activity

**Figure 1:**
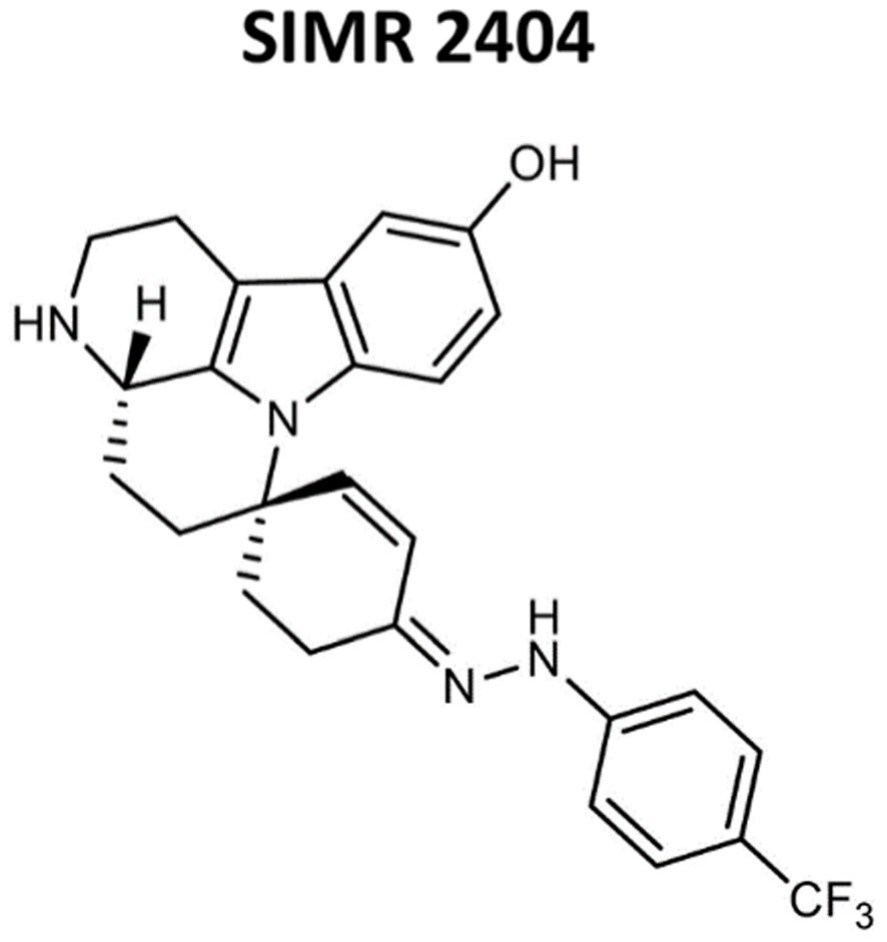
Chemical structures of compound SIMR 2404.

We evaluated the antimicrobial activity of new compounds developed in our laboratory (Altel et al., 2021; Srinivasulu et al., 2021) for their ability to inhibit the growth of Gram-positive and Gram-negative MDR pathogens. For Gram-positive bacteria, we tested three clinical MRSA strains and three reference ATCC strains including MRSA-ATCC700699 (vancomycin-intermediate *S. aureus*, VISA) (Supplementary Table S1). Tested Gram-negative bacteria were ATCC reference strains of *E. coli*, *K. pneumoniae*, *A. baumannii*, and *P. aeruginosa*. All six compounds showed growth inhibition against all tested MRSA strains with a minimum inhibitory concentration (MIC) ranging between 2–8 μg/mL (Supplementary Table S2). Against *E. coli* and *A. baumannii*, all six compounds showed growth inhibition at an MIC range between 8–64 μg/mL (Supplementary Table S3). None of the tested compounds showed activity against *K. pneumoniae* or *P. aeruginosa* (Data not shown; Table 1 for compound SIMR 2404). Based on these results, tested compounds were more active against Gram-positive bacteria compared to Gram-negative bacteria. We chose compound SIMR 2404 for further investigation. The chemical structure and MIC values for compound SIMR 2404 are shown in Table 1 and Figure 1.

**Table 1:** MICs (μg/mL) for compound SIMR 2404 against relevant multidrug-resistant bacteria.

Bacteria	SIMR 2404 MIC (μg/mL)
MRSA-ATCC33591	2
MRSA-ATCC700699 (VISA)	2
*Acinetobacter baumannii* ATCC 19606	8
*Acinetobacter baumannii* ATCC BAA 1605	32
*E. coli* CDC-AR-0346	64
*E. coli* ATCC BAA 2469	16
*Klebsiella Pneumonia* ATCC BAA-2146	Not Active
*Pseudomonas aeruginosa* ATCC 27853	Not Active

### Compound SIMR 2404 can rapidly kill MDR bacteria

**Figure 2:**
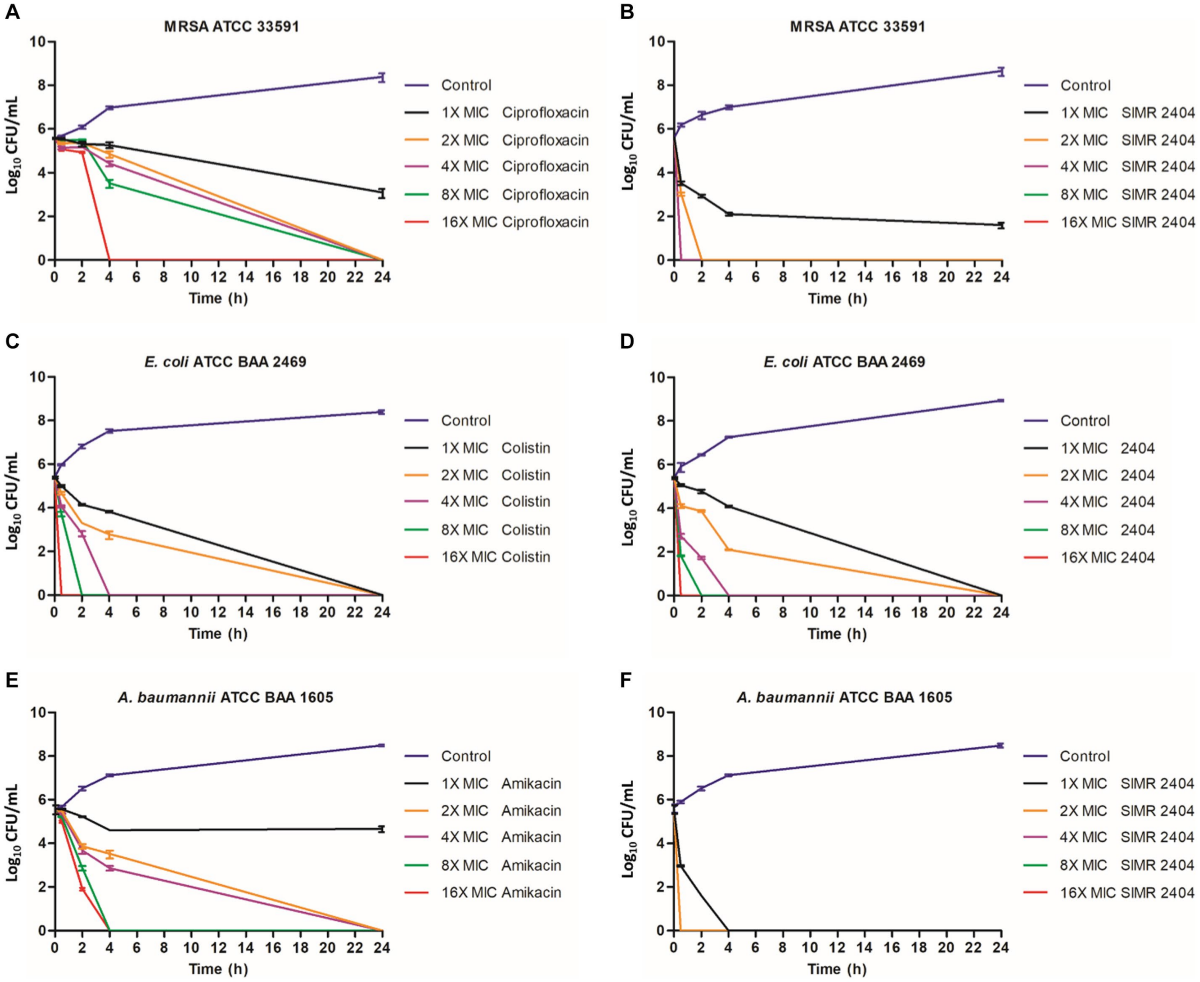
Killing kinetics of the SIMR 2404 against exponentially growing cells. 0.5 × 10^−5^ CFU/mL logarithmic phase bacterial cultures were exposed to compounds SIMR 2404 or control antibiotic. **(A)** MRSA ATCC 33591 treated with ciprofloxacin. **(B)** MRSA ATCC 33591 treated with SIMR 2404. **(C)** *E. coli* ATCC BAA 2469 treated with colistin. **(D)** *E. coli* ATCC BAA 2469 treated with SIMR 2404. **(E)** *A. baumannii* ATCC BAA 1605 treated with amikacin. **(F)** *A. baumannii* ATCC BAA 1605 treated with SIMR 2404. Serial dilution and CFU counts were used to determine viable counts.

**Figure 3:**
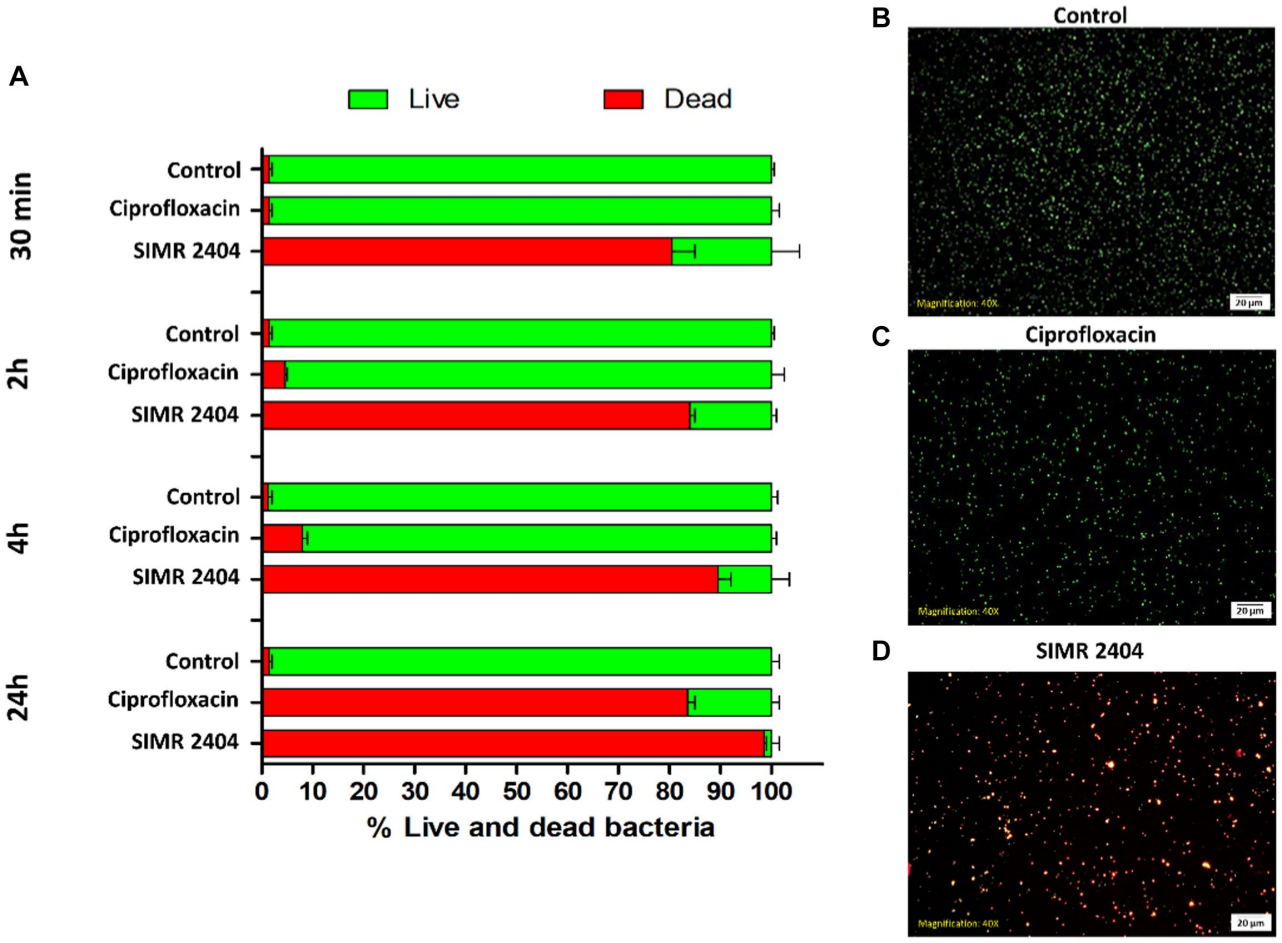
Live/Dead fluorescence assay of MRSA ATCC 33591 treated with SIMR 2404. **(A)** Exponential MRSA ATCC 33591 cultures (~10^7^ CFU/mL) treated with compounds SIMR 2404 or control antibiotic ciprofloxacin at 2XMIC were stainined with membrane permeable SYTO-9 (green) and membrane impermeable propidium iodide (red). The percentage of live cells (green) and dead cells (red or yellow) was calculated at indicated time points. The results are the average of three independent experiments. **(B–D)** Representative fluorescence microscopy images after 30 min of treatment. **(B)** Growth control. **(C)** Control antibiotic ciprofloxacin. **(D)** Compound SIMR 2404.

To evaluate the killing kinetics of compound SIMR 2404, a time-killing assay of exponentially growing (log phase) bacterial cultures was performed. At 16 × MIC of ciprofloxacin, which targets DNA replication, MRSA cultures were eradicated within 4 h (Figure 2A). This is in sharp contrast to compound SIMR 2404 which can rapidly eradicate MRSA cultures within 30 min at a lower concentration of 4 × MIC (Figure 2B). For *E. coli*, colistin, which targets bacterial membranes, was used as a control antibiotic and showed fast killing kinetics (Figure 2C). Surprisingly, compound SIMR 2404 showed similar rapid killing kinetics as colistin (Figure 2D). For *A. baumanii*, treatment with amikacin, which targets protein synthesis, was able to eradicate cultures at 8 × MIC after 4 h (Figure 2E). On the other hand, compound SIMR 2404 rapidly eradicates *A. baumanii* within only 30 min at a lower concentration of 2 × MIC (Figure 2F). Next, we examined the killing activity of compound SIMR 2404 against MRSA using live/dead staining and fluorescent microscopy. SYTO-9 is a green, fluorescent stain that penetrates cells with intact membranes, whereas the red, fluorescent stain, propidium iodide (PI), can only penetrate cells with damaged membranes. Therefore, live bacteria with intact membranes will fluoresce green while dead bacteria with compromised membranes will fluoresce red or yellow (Asadishad et al., 2011). Actively growing cultures of MRSA treated with compound SIMR 2404 showed ~84% dead cells after only 30 min of treatment (Figure 3). On the other hand, for the same time point of 30 min, the control antibiotic ciprofloxacin showed much slower killing activity of only ~2% (Figure 3). The slow killing activity of ciprofloxacin was also evident at 2 h and 4 h which was less than 10% (Figure 3). Only after a prolonged treatment of 24 h, ciprofloxacin was able to achieve a death rate of ~82% compared to SIMR 2404 which had a killing rate of ~99% for the same timepoint (Figure 3). Taken together, our data indicate that compared to the conventional antibiotics ciprofloxacin and amikacin, compound SIMR 2404 had a faster killing activity against MDR bacteria (Figures 2, 3). On the other hand, treatment with colistin or SIMR 2404 both showed fast killing activity (Figure 2). The data for the killing kinetics study are available in the Supplementary Tables S4–S6, and the ability of SIMR 2404 to eradicate MDR cultures within 30 min is presented in Supplementary Figure S2.

### Compounds SIMR 2404 eradicates MRSA persisters

**Figure 4:**
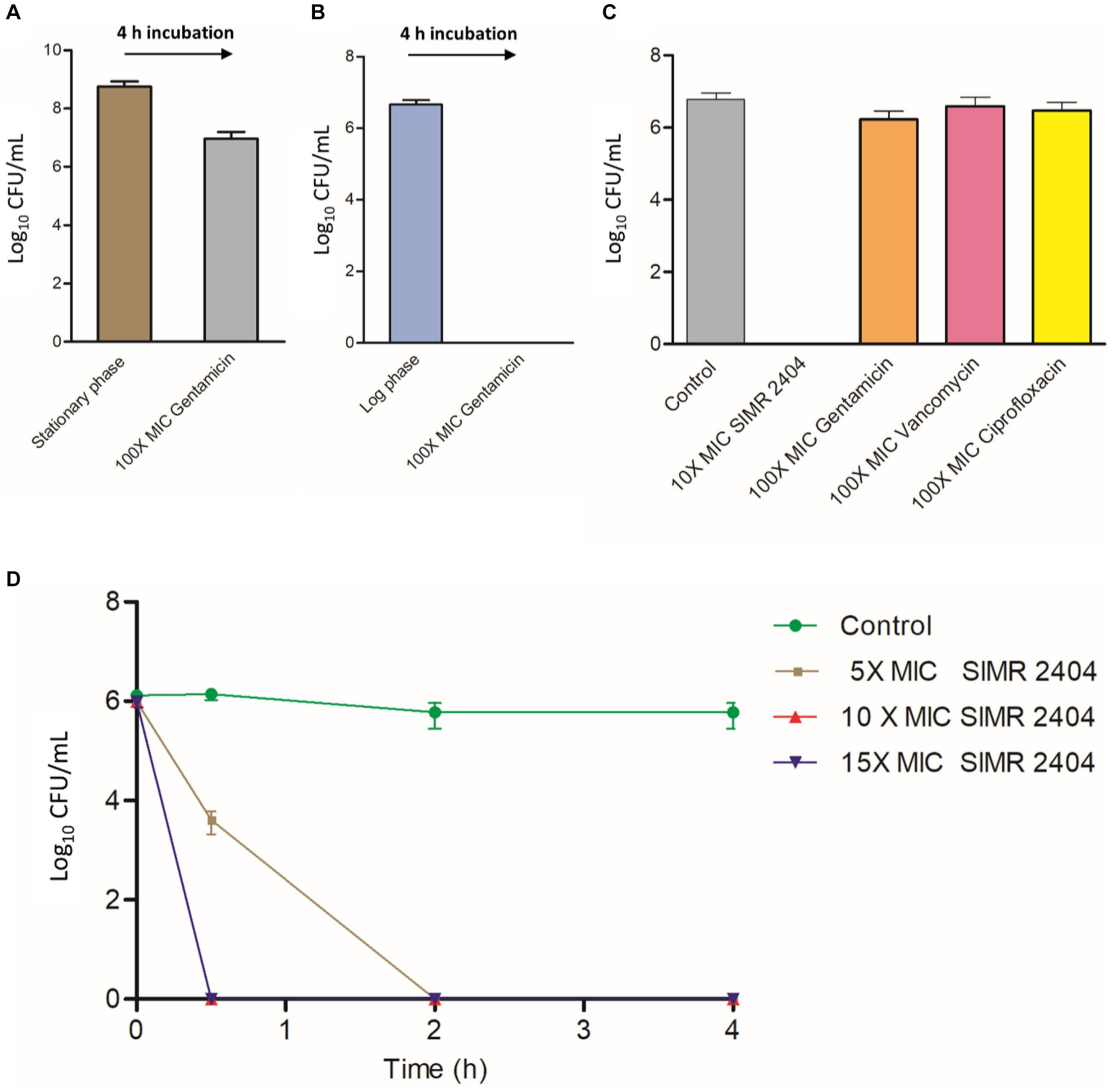
Killing kinetics of compound SIMR 2404 against persister MRSA ATCC 33591. **(A)** Preparation of MRSA ATCC 33591 persisters. Stationary phase overnight cultures of MRSA were treated with 100 × MIC gentamicin (250 μg/mL) for 4 h and viable persister counts were determined. **(B)** Gentamicin killing of log phase MRSA cultures treated with 100 × MIC gentamicin for 4 h. **(C)** Persister MRSA ATCC 33591 cells were adjusted to ~10^7^ CFU/mL and treated with compound SIMR 2404 or control antibiotics at indicated concentrations for an additional 4 h. **(D)** Killing kinetics of compounds SIMR 2404 against persister MRSA.

Next, we evaluated the ability of compound SIMR 2404 to kill MRSA persisters which are tolerant to conventional antibiotics. During the stationary phase, *S. aureus* develops a high number of persister cells which can be readily isolated by treating antibiotic-susceptible bacteria with a very high concentration of antibiotic for around 4 h (Lewis, 2005, 2010; Kim et al., 2016). We used MRSA-ATCC 33591 for persister experiments since it can develop persisters during the stationary phase (Li et al., 2020) and had low MIC to the antibiotic gentamicin (Supplementary Table S2). Stationary phase MRSA-ATCC 33591 treated with 100 × MIC of gentamicin for 4 h remained mostly viable (Figure 4A), unlike actively growing (log phase) cultures which were eradicated upon the same treatment (Figure 4B). Treatment of MRSA persisters prepared in (Figure 4A) with 10 × MIC of compound SIMR 2404, resulted in complete eradication of viable cells (Figure 4C). On the other hand, treatment with 100 × MIC of gentamicin, ciprofloxacin, or vancomycin showed virtually no antibacterial effects against persister MRSA (Figure 4C). Next, we performed time-kill experiments to evaluate the anti-persister activity of compound SIMR 2404. Within 2 h of treatment at 5 × MIC, compound SIMR 2404 was able to eradicate persister culture (Figure 4D). At 10 × MIC and 15 × MIC, eradication of persisters occurred faster and was achieved within only 30 min (Figure 4D).

### Evolution of resistance against compound 2404

**Figure 5:**
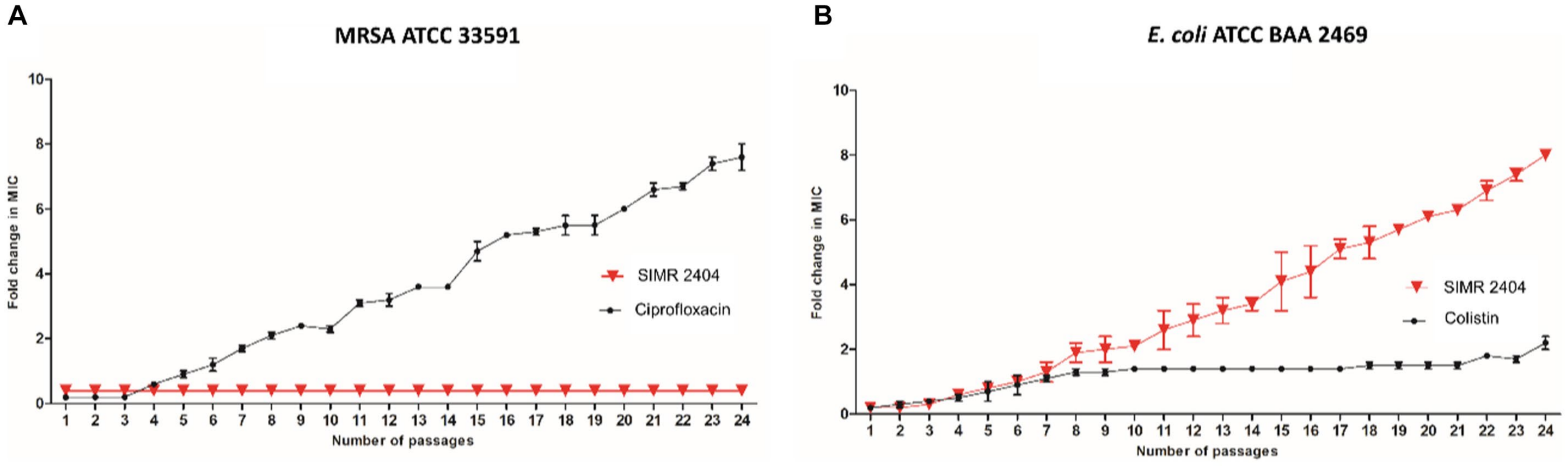
Multistep resistance evolution by serial passaging at sub-MIC levels. MRSA ATCC 33591 or *E. coli* ATCC BAA 2469 were grown at sub-MIC levels of **SIMR 2404** or indicated control antibiotic. Cultures that showed visible growth at the highest concentration were used the next day to inoculate media at increasing increments of the indicated compound or antibiotic. **(A)** Evolution of resistance of MRSA ATCC 33591 against SIMR 2404 or control antibiotic ciprofloxacin. **(B)** Evolution of resistance of *E. coli* ATCC BAA 2469 against SIMR 2404 or control antibiotic colistin.

To evaluate the ability of bacteria to develop resistance to compound SIMR 2404, we performed multistep resistance evolution experiments. For MRSA, serial dilution at increasing concentrations of ciprofloxacin resulted in the evolution of resistant cells which appeared within five passages (Figure 5A). However, no resistance was observed against compound SIMR 2404 even after 24 passages (Figure 5A). On the other hand, *E. coli* was able to develop resistance to compound SIMR 2404 after seven passages but failed to develop resistance to the control antibiotic colistin (Figure 5B). These data indicate that MRSA lacks the ability to develop resistance to compound SIMR 2404, unlike *E. coli* which can become resistant following repeated exposures.

### Toxicity profile of compound 2404 against normal human fibroblast cells

**Figure 6:**
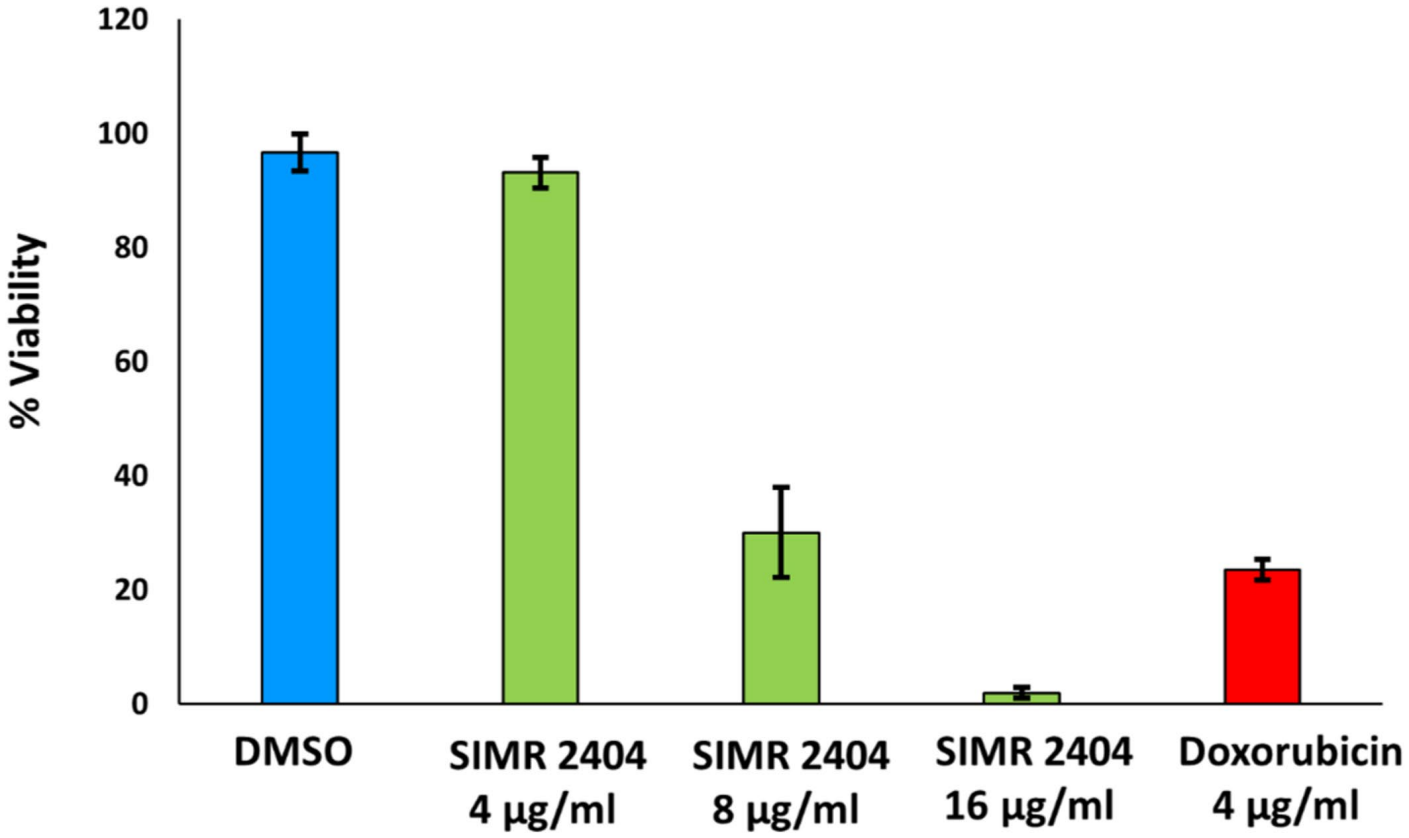
Cytotoxic effect against normal human fibroblast cells (F180). Survival of F180 cells was measured using sulforhodamine B (SRB) assay in response to treatment of compounds at indicated concentrations. DMSO was used as a negative control while doxorubicin was used as a positive control of cytotoxicity.

The toxicity profile of compound SIMR 2404 was evaluated against normal human fibroblast cells (F180) using the sulforhodamine B (SRB) assay. At 4 μg/mL, SIMR 2404 showed no cytotoxic effect compared to the negative control DMSO (Figure 6). However, at a concentration of 8 μg/mL and above, SIMR 2404 showed significant toxicity similar to the positive cytotoxic control doxorubicin (Figure 6). Given that the MIC of SIMR 2404 against MRSA was 2 μg/mL, these results suggest that this compound might be a safe candidate for further studies against active MRSA; however, it is an unlikely candidate against Gram-negative bacteria.

## Discussion

The use of “Complexity to diversity” schemes for the chemical synthesis of nature-inspired molecules is a promising approach for the development of underrepresented chemical space with unique biological activities (Cj et al., 2012; Eder et al., 2014; Kim et al., 2014). We have previously described the synthesis schemes of nature-inspired molecules using this approach, and in this work, we evaluated the antimicrobial activity of a few of these molecules against MDR pathogens (Srinivasulu et al., 2021). Our results show that these molecules have potent antimicrobial activity against the Gram-positive bacteria methicillin-resistant *Staphylococcus aureus* (MRSA) and vancomycin-intermediate *Staphylococcus aureus* (VISA). These molecules also show moderate antimicrobial activity against Gram-negative MDR pathogens such as *E. coli* and *A. baumannii* but not against *P. aeruginosa* and *K. pneumoniae*. The reduced activity against Gram-negative bacteria is likely due to the permeability barrier of the outer membrane which is only present in Gram-negative bacteria (Ghai and Ghai, 2018). The lack of activity against *P. aeruginosa* and *K. pneumoniae* could be a result of the differences in outer membrane permeability due to the differences in lipopolysaccharide structures (Lam et al., 2011; Choi et al., 2020) or could be due to the presence of multidrug efflux pumps (Du et al., 2018; Ni et al., 2020). Further studies are needed to elucidate the various resistance profiles of Gram-negative bacteria against SIMR 2404 as well as the increased resistance to Gram-negative bacteria compared to Gram-positive bacteria.

Compound SIMR 2404 showed fast killing rates against both Gram-positive and Gram-negative bacteria when compared with conventional antibiotics with the exception of colistin. Colistin is considered a last resort drug against Gram-negative bacteria, and its main mechanism of action involves binding to bacterial membranes leading to cell lysis (Biswas et al., 2012; Sabnis et al., 2021). Given that the killing kinetics of both colistin and compound SIMR 2404 against *E. coli* were very similar, we speculate that SIMR 2404 might kill bacteria by damaging their membranes. This is further supported by fluorescent microscopy using live/dead staining which showed that compound SIMR 2404 can induce rapid membrane damage in MRSA within only 30 min.

In addition to its resistance to antibiotics, MRSA can form persister cells that are tolerant to conventional antibiotics (Lewis, 2005, 2010). Eradicating drug-tolerant persisters, although difficult, has been achieved through metabolic activation (Allison et al., 2011), targeting bacterial components which are independent of active metabolism such as bacterial membranes (Kim et al., 2018a,b,c), or DNA crosslinking (Kwan et al., 2015; Chowdhury et al., 2016). Compound SIMR 2404 was found to kill persister MRSA within 4 h unlike the conventional antibiotics gentamicin, ciprofloxacin, and vancomycin. Furthermore, in time-kill experiments, increasing the concentration of compound SIMR 2404 led to faster eradication of MRSA persisters. While the antimicrobial mechanism of action of compound SIMR 2404 remains to be elucidated, there are three lines of evidence that strongly indicate that SIMR 2404 kills bacteria by damaging their membranes. First, SIMR 2404 can rapidly kill metabolically inactive persisters, which are usually only killed through membrane damage, in a concentration-dependent manner. Second, it can kill actively dividing bacteria at a rate much faster than ciprofloxacin which targets DNA replication, or amikacin which targets protein synthesis, but it is equally fast to the killing kinetics of the membrane targeting antibiotic colistin. Finally, microscopic analysis in the live/dead experiments showed that treatment with SIMR 2404 results in fast staining by propidium iodide (PI) which can only penetrate cells with damaged or compromised membranes. While all these lines of evidence strongly suggest that the mechanism of action of SIMR 2404 is likely mediated by bacterial membrane disruption, more studies are needed to test this hypothesis. When compound SIMR 2404 was studied for the evolution of resistance, MRSA failed to develop resistance despite repeated exposure; however, *E. coli* was able to develop resistance. While we currently do not know the mechanisms or mutations behind resistance development in *E. coli*, current work in our laboratory using whole genome sequencing and comparative genomics with the parent-sensitive strain is underway to identify these mutations. With regard to the toxicity profile, compound SIMR 2404 showed no toxic effect at 4 μg/mL which is twice the MIC concentration against MRSA, but was toxic at a concentration of 8 μg/mL and higher. Based on these results, SIMR 2404 does not appear ideal as a candidate against Gram-negative bacteria given their high MIC values, toxic profile, and ability to develop resistance. However, it is worth mentioning that future studies investigating these downsides could potentially help to alleviate them. For example, the study that demonstrated the activity of nTZDpa against persister MRSA also found significant toxicity against mammalian cells (Kim et al., 2018b). However through synthetic chemistry guided by molecular dynamics simulations, selective and less toxic derivatives of nTZDpa were generated (Kim et al., 2018b). A similar approach was employed on the synthetic retinoid CD437 to produce an analog that retained anti-persister activity against MRSA but improved its cytotoxicity profile against mammalian cells (Kim et al., 2018c). In conclusion, our data indicate that compound SIMR 2404 is a promising candidate for future investigation as a candidate antimicrobial against MRSA.

## Data availability statement

The original contributions presented in the study are included in the article/Supplementary material, further inquiries can be directed to the corresponding author.

## Ethics statement

Ethical approval was not required for the studies on humans in accordance with the local legislation and institutional requirements because only commercially available established cell lines were used.

## Author contributions

MH: Conceptualization, Funding acquisition, Investigation, Methodology, Writing – original draft, Writing – review & editing. FA-M: Methodology, Writing – original draft, Writing – review & editing. VS: Methodology, Writing – original draft. AS: Investigation, Methodology, Writing – original draft. VM: Methodology, Writing – original draft. WR: Investigation, Methodology, Writing – original draft. RE-A: Writing – review & editing. TA-T: Conceptualization, Funding acquisition, Investigation, Methodology, Supervision, Writing – original draft, Writing – review & editing.
