# Supplementary material for: Antimicrobial activity of nature-inspired molecules against multidrug-resistant bacteria
Source: Front Microbiol. 2024 Jan 22;14:1336856. doi: 10.3389/fmicb.2023.1336856 (PMC10838778; doi:10.3389/fmicb.2023.1336856)
Supplement: Supplementary file 1 [file Data_Sheet_1.docx]

**Antimicrobial activity of nature-inspired molecules against multidrug resistant bacteria**

**Mohamad Hamad^1,2^*****, Farah Al-Marzooq^3^, Ashna Sulaiman^1^, Varsha Menon^1^, Wafaa S. Ramadan^1^, Raafat El-Awady ^1,4^, and Taleb H. Al-Tel ^1,4^**

^1^Sharjah Institute for Medical Research, University of Sharjah, Sharjah, United Arab Emirates

^2^ College of Health Sciences, University of Sharjah, Sharjah, United Arab Emirates

^3^College of Medicine and Health Sciences, UAE University, Al Ain, United Arab Emirates

^4^College of Pharmacy, University of Sharjah, Sharjah, United Arab Emirates

**Supplementary Table 1S. List of bacterial strains used in this study.**

| **Bacteria** | **Source** | **Relevant characteristic** |
| --- | --- | --- |
| *S. aureus* (MRSA-1*) | Clinical isolate | Gram positive/MDR |
| *S. aureus* (MRSA-2*) | Clinical isolate | Gram positive/MDR |
| *S. aureus* (MRSA-3*) | Clinical isolate | Gram positive/MDR |
| *S. aureus* (MRSA-ATCC 33591) | Reference strain (ATCC) | Gram positive/MDR |
| *S. aureus* (MRSA-ATCC 33592) | Reference strain (ATCC) | Gram positive/MDR |
| *S. aureus* (MRSA- ATCC 700699) | Reference strain (ATCC) | Gram positive/MDR/VISA (Intermediate resistance to vancomycin) |
| *E. coli* (ATCC 25922) | Reference strain (ATCC) | Gram negative bacteria |
| *E. coli* (ATCCBAA2469) | Reference strain (ATCC) | Gram negative bacteria/MDR/(NDM-1 carbapenem-resistant) |
| *E. coli* (CDC-AR-0346) | Reference strain (CDC) | Gram negative bacteria/MDR/(ESBL, Colistin resistant) |
| *P. aeruginosa* (ATCC 27853) | Reference strain (ATCC) | Gram negative bacteria |
| *A. baumannii* (ATCC 19606) | Reference strain (ATCC) | Gram negative bacteria/MDR |
| *A. baumannii* (ATCC BAA1605) | Reference strain (ATCC) | Gram negative bacteria/MDR/(Carbapenem-resistant) |
| *K. pneumoniae* (ATCC BAA- 2146) | Reference strain (ATCC) | Gram negative bacteria/MDR |

* Reference: (Hamad et al., 2022)


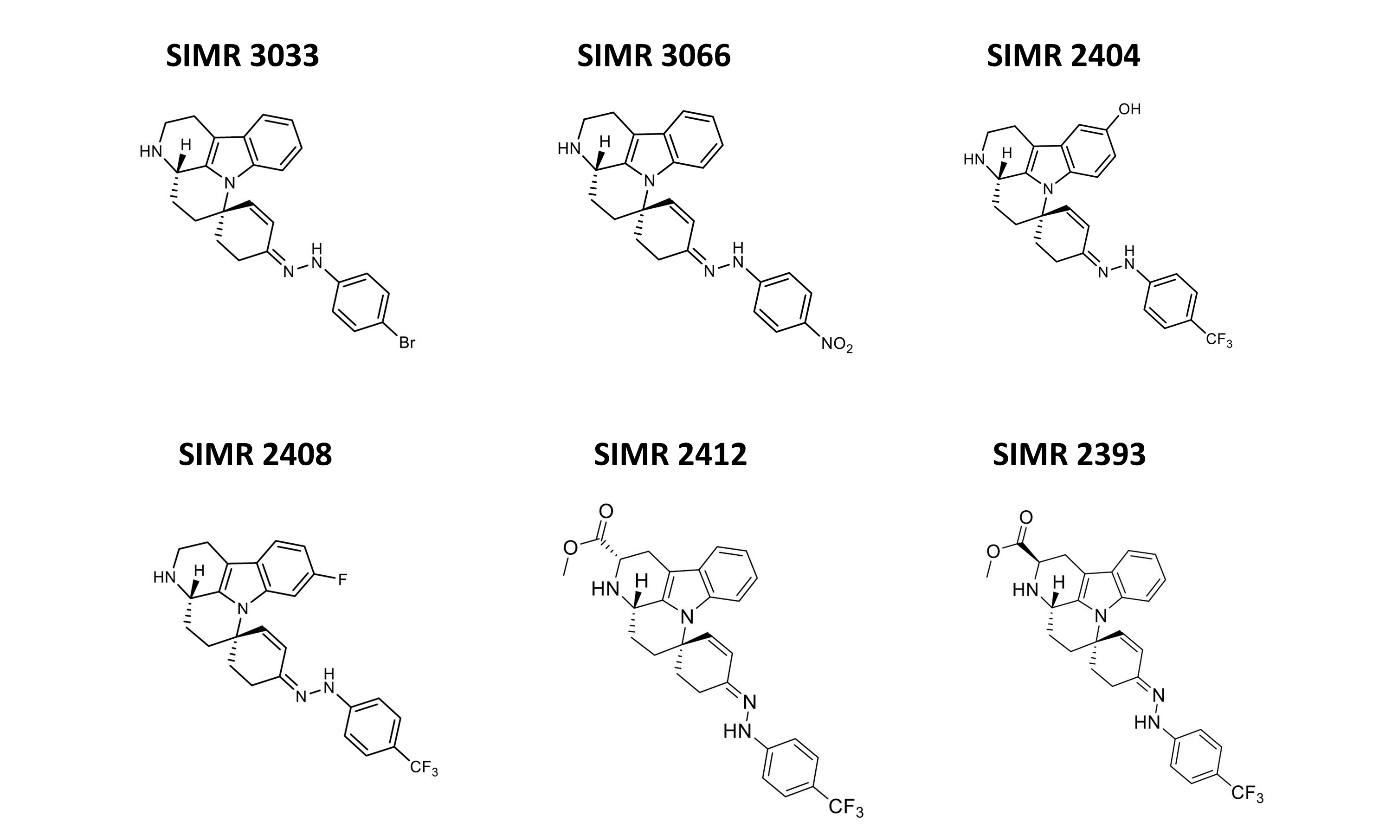


**Figure 1S. Chemical structure of test compounds.**

**Table 2S. MIC values (µg/mL) for the selected compounds Gram-positive, MDR MRSA strains:**

| **Compound** | **MIC (μg/mL)** | | | | | |
| --- | --- | --- | --- | --- | --- | --- |
|  | **MRSA-1** (**Clinical isolate)** | **MRSA-2**  (**Clinical isolate)** | **MRSA-3**  (**Clinical isolate)** | **MRSA-ATCC33591** | **MRSA-ATCC33592** | **MRSA-ATCC700699 (VISA)*** |
| **SIMR 3033** | 2 | 2 | 2 | 4 | 4 | 4 |
| **SIMR 3066** | 2 | 2 | 2 | 4 | 4 | 4 |
| **SIMR 2404** | 2 | 2 | 2 | 2 | 2 | 2 |
| **SIMR 2408** | 2 | 4 | 4 | 4 | 4 | 2 |
| **SIMR 2412** | 2 | 2 | 2 | 2 | 2 | 2 |
| **SIMR 2393** | 8 | 8 | 8 | 8 | 8 | 8 |
| **Vancomycin** | 1 | 1 | 1 | 1 | 1 | 8 |
| **Gentamicin** | 50 | 0.625 | 0.625 | 2.5 | >500 | 500 |
| **Amikacin** | 16 | 8 | 2 | 50 | 50 | 100 |
| **Ciprofloxacin** | 32 | 16 | 2 | 0.3 | 0.3 | 16 |

*(Intermediate resistance to vancomycin)

**Table 3S. MIC values (µg/mL) for the selected compounds tested on 5 Gram-negative bacterial strains.**

| **Compound** | **MIC (μg/mL)** | | | | |
| --- | --- | --- | --- | --- | --- |
|  | ***E. coli* ATCC25922 *** | ***E. coli***  **ATCC BAA2469 (NDM-1 carbapenem-resistant)** | ***E. coli***  **CDC-AR-0346**  **(ESBL +, colistin resistant)** | ***Acinetobacter baumannii***  **ATCC 19606*** | ***Acinetobacter baumannii***  **ATCC BAA1605 (carbapenem-resistant)** |
| **SIMR 3033** | 8 | 16 | 16 | 8 | 32 |
| **SIMR 3066** | 8 | 16 | 64 | 8 | 64 |
| **SIMR 2404** | 8 | 16 | 16 | 8 | 32 |
| **SIMR 2408** | 16 | 16 | 32 | 8 | 32 |
| **SIMR 2412** | 8 | 16 | 16 | 8 | 32 |
| **SIMR 2393** | 16 | 25 | 25 | 16 | 64 |
| **Meropenem** | <0.02 | 25 | <0.2 | 0.6 | 16 |
| **Colistin** | 0.2 | 1.25 | 8 | 1.25 | 1.25 |
| **Gentamicin** | 0.625 | >1000 | >1000 | 10 | 125 |
| **Amikacin** | 2.5 | >1000 | >1000 | 10 | 1.25 |
| **Ciprofloxacin** | <0.02 | 100 | 16 | 0.625 | 50 |
| **Cefotaxime** | 0.03 | 250 | >1000 | 20 | 1000 |
| **Ceftriaxone** | 0.03 | 500 | >1000 | 20 | >1000 |

* Not MDR. + Resistant to first, second, third and fourth generation cephalosporines.

**Table 4S. Mean CFU/mL of MRSA-ATCC33591 treated with ciprofloxacin or SIMR 2404 overtime.**

| Compound/antibiotic | Time 0 h | Time 0.5 h | Time 2 h | Time 4 h | Time 24 h |
| --- | --- | --- | --- | --- | --- |
| None | 366666 | 473333 | 1200000 | 9333333 | 246000000 |
| Ciprofloxacin 1XMIC | 366666 | 346666 | 340000 | 800000 | 1253 |
| Ciprofloxacin 2XMIC | 366666 | 220000 | 233333 | 73333 | 0 |
| Ciprofloxacin 4XMIC | 366666 | 140000 | 153333 | 26666 | 0 |
| Ciprofloxacin 8XMIC | 366666 | 300000 | 113333 | 3333 | 0 |
| Ciprofloxacin 16XMIC | 366666 | 120000 | 86666 | 0 | 0 |
| SIMR 2404 1XMIC | 366666 | 3333 | 866 | 126 | 40 |
| SIMR 2404 2XMIC | 366666 | 1066 | 0 | 0 | 0 |
| SIMR 2404 4XMIC | 366666 | 0 | 0 | 0 | 0 |
| SIMR 2404 8XMIC | 366666 | 0 | 0 | 0 | 0 |
| SIMR 2404 16XMIC | 366666 | 0 | 0 | 0 | 0 |

**Table 5S. Mean CFU/mL of *E. coli* ATCC BAA 2469 treated with colistin or SIMR 2404 overtime.**

| Compound/antibiotic | Time 0 h | Time 0.5 h | Time 2 h | Time 4 h | Time 24 h |
| --- | --- | --- | --- | --- | --- |
| None | 246666 | 633333 | 6666666 | 33333333 | 280000000 |
| Colistin 1XMIC | 246666 | 100000 | 14000 | 6666 | 0 |
| Colistin 2XMIC | 246666 | 46666 | 2000 | 600 | 0 |
| Colistin 4XMIC | 246666 | 11333 | 666 | 0 | 0 |
| Colistin 8XMIC | 246666 | 5333 | 0 | 0 | 0 |
| Colistin 16XMIC | 246666 | 0 | 0 | 0 | 0 |
| SIMR 2404 1XMIC | 246666 | 1133333 | 106666 | 12000 | 0 |
| SIMR 2404 2XMIC | 246666 | 12666 | 7333 | 126 | 0 |
| SIMR 2404 4XMIC | 246666 | 533 | 60 | 0 | 0 |
| SIMR 2404 8XMIC | 246666 | 66 | 0 | 0 | 0 |
| SIMR 2404 16XMIC | 246666 | 0 | 0 | 0 | 0 |

**Table 6S. Mean CFU/mL of *A. baumannii* ATCC BAA 1605** **treated with amikacin or SIMR 2404 overtime.**

| Compound/antibiotic | Time 0 h | Time 0.5 h | Time 2 h | Time 4 h | Time 24 h |
| --- | --- | --- | --- | --- | --- |
| None | 466666 | 666666 | 3333333 | 13333333 | 306666666 |
| Amikacin 1XMIC | 466666 | 366666 | 166666 | 40000 | 46666 |
| Amikacin 2XMIC | 466666 | 306666 | 7333 | 3333 | 0 |
| Amikacin 4XMIC | 466666 | 233333 | 4666 | 733 | 0 |
| Amikacin 8XMIC | 466666 | 166666 | 600 | 0 | 0 |
| Amikacin 16XMIC | 466666 | 100000 | 80 | 0 | 0 |
| SIMR 2404 1XMIC | 466666 | 933 | 126 | 0 | 0 |
| SIMR 2404 2XMIC | 466666 | 0 | 0 | 0 | 0 |
| SIMR 2404 4XMIC | 466666 | 0 | 0 | 0 | 0 |
| SIMR 2404 8XMIC | 466666 | 0 | 0 | 0 | 0 |
| SIMR 2404 16XMIC | 466666 | 0 | 0 | 0 | 0 |


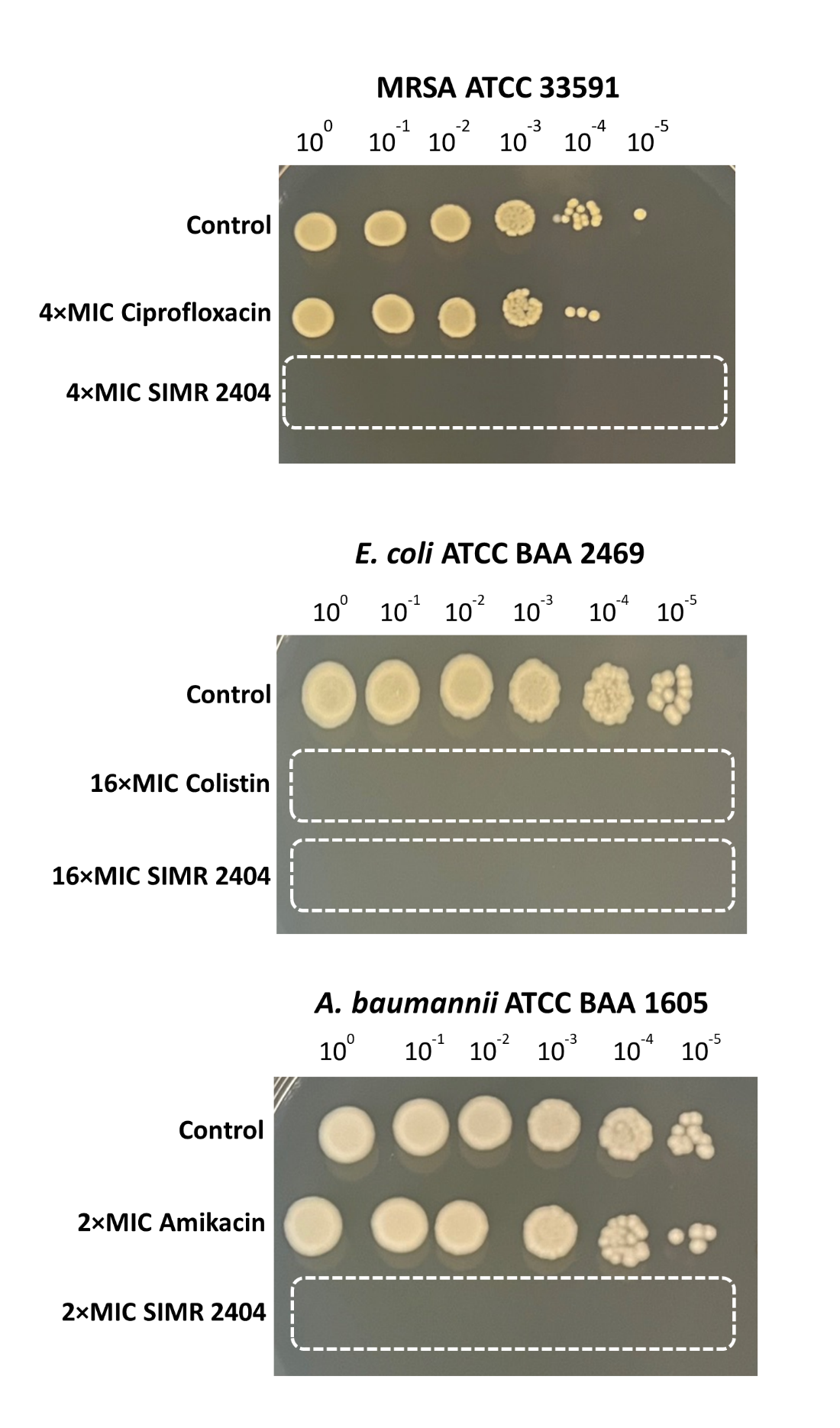


**Figure 2S. Bacterial growth after treatment with control antibiotics or SIMR 2404 at indicated concentration for 30 minutes. Lack of bacterial growth is highlighted by dotted box.**

**Reference:**

Hamad, M., Al-Marzooq, F., Srinivasulu, V., Omar, H.A., Sulaiman, A., Zaher, D.M., Orive, G., and Al-Tel, T.H. (2022). Antibacterial Activity of Small Molecules Which Eradicate Methicillin-Resistant Staphylococcus aureus Persisters. *Front Microbiol* 13**,** 823394 DOI: <https://doi.org/10.3389/fmicb.2022.823394>.
